# Detachment of Breast Tumor Cells Induces Rapid Secretion of Exosomes Which Subsequently Mediate Cellular Adhesion and Spreading

**DOI:** 10.1371/journal.pone.0024234

**Published:** 2011-09-06

**Authors:** Rainelli B. Koumangoye, Amos M. Sakwe, J. Shawn Goodwin, Tina Patel, Josiah Ochieng

**Affiliations:** Department of Biochemistry and Cancer Biology, Meharry Medical College, Nashville, Tennessee, United States of America; The University of Kansas Medical Center, United States of America

## Abstract

Exosomes are nano-vesicles secreted by a wide range of mammalian cell types. These vesicles are abundant in serum and other extracellular fluids and contain a large repertoire of proteins, mRNA and microRNA. Exosomes have been implicated in cell to cell communication, the transfer of infectious agents, and neurodegenerative diseases as well as tumor progression. However, the precise mechanisms by which they are internalized and/or secreted remain poorly understood. In order to follow their release and uptake in breast tumor cells in real time, cell-derived exosomes were tagged with green fluorescent protein (GFP)-CD63 while human serum exosomes were rhodamine isothiocynate-labeled. We show that detachment of adherent cells from various substrata induces a rapid and substantial secretion of exosomes, which then concentrate on the cell surfaces and mediate adhesion to various extracellular matrix proteins. We also demonstrate that disruption of lipid rafts with methyl-beta-cyclodextrin (MβCD) inhibits the internalization of exosomes and that annexins are essential for the exosomal uptake mechanisms. Taken together, these data suggest that cellular detachment is accompanied by significant release of exosomes while cellular adhesion and spreading are enhanced by rapid uptake and disposition of exosomes on the cell surface.

## Introduction

Exosomes are small nanovesicles (30–100 nm) that originate from the inward budding of an endosome's limiting membrane into its lumen, giving rise to endosomes containing multiple intra-luminal vesicles and therefore termed multivesicular body (MVB). The outer membranes of MVBs can fuse with the plasma membrane and release their intraluminal vesicles to the extracellular milieu as exosomes [1], [2]. Even though exosomes were described more than two decades ago, it is only in the last three years that thorough mechanistic studies of their functional roles commenced in cancer. These recent studies have suggested that exosomes take part in key physiological processes such as cell-cell communication, cellular adhesion, migration, invasion, angiogenesis and growth of tumor cells [3], [4], [5], [6]. Therefore, they can no longer be considered simply as garbage bags for throwing out unwanted protein cargo from the cell as originally suggested [7], necessitating a recalibration of our understanding and of their potential functions in physiological processes.

Exosomes have been shown to mediate the adhesion of breast epithelial cells in culture [6]. Adhesion is exceedingly important not only in cancer biology but other pathological conditions including cardiovascular disorders [8], [9]. It is generally assumed that integrins on the cell surface are the major if not the only players in extracellular matrix adhesion [10]. Recent studies involving tumor cells, however, show that the process may be more complicated, involving players other than integrins. Whereas integrins require the presence of manganese or magnesium for optimal activity [11], exosomal mediated adhesion and spreading can be affected by calcium (unpublished information-J.O.) Furthermore, we recently reported that in breast carcinoma cells, fetuin-A and calcium ions may be just as important as integrins in mediating adhesion dependent growth signaling mechanisms [12]. Intriguingly, platelet-derived microvesicles depleted of exosomes have also been shown to mediate cell to cell as well as cell to extracellular matrix (ECM) adhesion [13].

Due to multiple extracellular and intracellular physiological processes that can be attributed to exosomes, there is a need to define biochemical mechanisms that mediate secretion and uptake of these vesicles. For example, it is becoming increasingly clear that factors that impose stress on cells can mediate the secretion of exosomes [14]. Increases in intracellular calcium that can be induced by growth factors and ionophores have been shown to mediate secretion of exosomes [15]. It is feasible that spikes in intracellular calcium which occur for example when cells detach from the substrata could be responsible for both the constitutive and regulated secretion of exosomes. A number of studies utilizing labeled exosomes have reported rapid uptake of these vesicles by cells [16], [17]. Some studies have shown that exosomes are internalized via phagocytosis [18], while others suggest lipid raft domains [19]. Whereas it is believed that exosomal secretion and uptake is a means of intercellular communication including the exchange of microRNA and messenger RNA [20], there are potentially other reasons why cells and especially tumor cells uptake these vesicles.

The impetus for the present studies were the reports that galectin-3 (Gal-3) and other members of the family are secreted in exosomes [16], [21], [22], [23] and our previous report showing that Gal-3 secretion is increased in detached cells [24]. In order to define the processes of exosomal secretion and uptake, we stably transfected BT-549 breast cancer cells with green fluorescent protein (GFP)-tagged CD63 and followed the secretion and uptake of the GFP-labeled exosomes. CD63 is an integral membrane protein known to be predominantly associated with MVB/late endosomes and exosomal membranes [25], [26], [27]. Using this approach we report, that exosomes are rapidly secreted upon detachment of cells from substrata, a process that can be followed in live cells. Interestingly, upon attachment, exosomes are taken up by the adhered and spreading cells via membrane lipid rafts domains. Annexins, particularly AnxA2 and AnxA6 are required for the uptake mechanism. In addition, AnxA2 is associated with immobile raft domains and is likely to immobilize exosomes on the cell surface at specific adherent junctions.

## Materials and Methods

### Materials

Polyclonal antibodies to AnxA2, AnxA6, EEA1, CD71, LAMP1, HSP90, and CD63 were purchased from Santa Cruz Biotechnology Inc. (Santa Cruz, CA, USA). Monoclonal antibody to green fluorescent protein (GFP) was purchased from OriGene Technologies Inc. (Rockville, MD, USA). Alamar Blue, Alexa fluor 555-tagged cholera toxin B and Alexa fluor 568-tagged transferrin conjugates were purchased from Invitrogen (Carlsbad, CA, USA). All the other reagents were purchased from Sigma (St. Louis, MO, USA) unless otherwise stated.

### Cell culture

The breast carcinoma cell lines (BT-549) and MDA-MB-231 were purchased from ATCC (Manassas, VA). A subclone of BT-549 stably transfected with galectin-3 (BT-549Gal-3) was a gift from Dr. Avraham Raz of Karmanos Cancer Research Institute. These cells were propagated in culture medium (Dulbecco's modified Eagle's medium/nutrient F-12 (DMEM/F-12) supplemented with 10% heat-inactivated fetal bovine serum, 2 mmol/liter L-glutamine, 100 units/ml penicillin, and 50 units/ml streptomycin in a humidified 95% air and 5% CO_2_ incubator at 37°C). Where indicated, serum-free medium (SFM) consisted of DMEM/F-12 in which fetal bovine serum (FBS) was replaced with 0.1% bovine serum albumin (BSA).

### Expression of GFP-tagged CD63 in BT-549 breast cancer cells

Human CD63 cDNA in pCMV6-AC-GFP (pCMV6-GFP-CD63) was purchased from OriGene Technologies Inc. (Rockville, MD). BT-549 cells were grown overnight in six-well plates at 60 to 70% confluence, then transfected with 4 µg/well of purified pCMV6-GFP-CD63 by using Fugene6 reagent as recommended by the manufacturer (Roche Applied Science, Indianapolis, IN). Cells were selected in complete DMEM/F-12 medium containing 500 µg/mL geneticin (G418) for three weeks. G418 resistant and GFP positive cells were further isolated using fluorescence activated cell sorting (FACS), expanded and maintained in selection medium as long as the cells were in culture. The expression of GFP-CD63 in the selected cell lines herein referred to as BT-CD63 cells was verified by immunoblotting and immunofluorescence.

#### Purification of exosomes from adherent and non-adherent cells

BT-549 and BT-CD63 cells were cultured in exosome-free medium (complete medium depleted of FBS-derived exosomes) prepared as previously described [12]. The conditioned media were first centrifuged at 20,000 × g for 30 minutes to remove micro-vesicles and other cellular debris. The resultant supernatant was carefully collected, filtered through a 0.22- µm pore filter (Millipore) and the exosomes pelleted by ultracentrifugation at 100,000 x g for 1 hour at 4°C. The exosome-containing pellets were dissolved in HBSS containing 1 mM MgCl_2_ and CaCl_2_. For purification of exosomes in non-adherent (suspension) cells, BT-549 and BT-CD63 were grown as described above. Cells were then washed twice with HBSS, detached from the substrata using 2 mM EDTA and pelleted at 700 x g for 5 minutes. These were washed to remove EDTA and re-suspended in 5 mL of EFM containing 1 mM MgCl_2_ and CaCl_2_. Cells were subsequently incubated at 37°C for 2 hours. The culture supernatants were then centrifuged at 3000 x g for 5 minutes to remove any cells and then at 20,000 ×g for 15 minutes to remove micro-vesicles and other cellular debris. The final supernatant was carefully collected, passed through a 0.22- µm pore filter (Millipore) and exosomes were concentrated by ultracentrifugation at 100,000 x g for 1 hour at 4°C. The concentration of proteins was determined by the Bradford assay. To quantify GFP-CD63 labeled exosomes, BT-CD63 cells were grown in complete DMEM/F-12 medium in 10 cm plates for 24 hours. Cells were washed twice with PBS and the complete medium was replaced with 2% FBS DMEM/F-12 medium without phenol red and exosomes in the conditioned medium were transferred to 96 well plates and quantified by fluorescence spectroscopy following excitation at 488 nm and emission at 509 nm (BioTek). We previously demonstrated the purity of these exosomes by electron microscopy [6].

Further purification of exosomes, concentrated by differential velocity centrifugation as described above, was performed by density gradient centrifugation using sucrose step gradients (10% to 60%). The exosome-loaded gradients were centrifuged at 200, 000 x g for 4 hours at 4°C using the Sorvall M150 micro-ultracentrifuge and S120-AT2 rotor. Ten 120 µL fractions were collected from the top of the gradient. Aliquots (20 µL) of each fraction were then resolved in 4-12% SDS-PAGE and analyzed by Western blotting.

### Exosome uptake assays

Human serum exosomes were purified as previously described [6]. The purified exosomes were re-suspended in 200 µL of HBSS. GFP-CD63-labeled cell-derived exosomes were purified from culture supernatants of BT-CD63 cells. BT-CD63, BT-549 and BT-549 cells stably transfected with GFP-AnxA2 or GFP-AnxA6 cells were either grown in complete DMEM/F12 on microscope coverslips (adherent cultures) or in anchorage-independent mode on polyHEMA (Sigma) coated six-well plates. These were then incubated with GFP-CD63-labeled cell-derived exosomes (10 µg/ml) at 37°C for up to 3 hours. Cells growing on polyHEMA coated wells were then transferred to glass cover slips coated with poly-L-Lysine (Sigma) and allowed to attach for 20 minutes in complete medium. The cells were then washed three times with cold PBS, fixed in 3.7% paraformaldehyde for 15 minutes at room temperature, quenched with 0.1 M glycine in cold PBS for 30 minutes, washed three times with PBS, and mounted with ProLong antifade reagent (Invitrogen). Internalization of exosomes was observed under a laser scanning confocal microscope (Nikon A1R).

### Flow cytometry

BT-549 cells were pretreated without or with methyl-β-cyclodextrin (MβCD) (10 mM) for 1 hour, and then incubated with 10 µg/mL of GFP-labeled exosomes in PBS containing 0.5% BSA. Cells were then fixed with 1% paraformaldehyde, quenched with 0.1 M glycine in PBS, and permeabilized with PBS/0.5% BSA, 0.02% Triton X-100. In the exosomal uptake studies, BT-549, BT-A2-sh and BT-A6-sh, cells were incubated with GFP-CD63 labeled exosomes diluted in PBS 0.5% BSA at 37°C for 30 minutes, fixed with 1% paraformaldehyde and quenched with PBS 0.1 M glycine (surface staining). For total staining (intracellular + surface staining) as described by Ostrowski et al [28], cells were incubated with GFP-CD63 exosomes then fixed with 1% paraformaldehyde, quenched with PBS 0.1 M glycine, permeabilized using PBS 0.5% BSA, 0.02% Triton X-100. Flow cytometry data was acquired on a FACS calibur and analyzed using FlowJo software.

### Cell attachment assays

The wells of 96-well plates were coated in quintuplets with fibronectin or laminin (10 µg/mL) in PBS. Prior to use, the coating solutions were discarded, and wells washed twice with PBS. BT-549 cells growing as adherent cultures in complete DMEM/F-12 were harvested using 2 mM EDTA, washed and re-suspended in EFM at 37°C for 2 hours to rid the cells of endogenous exosomes. Cells were then pelleted and re-suspended in serum-free DMEM/F-12 or serum-free DMEM/F-12 supplemented with exogenous exosomes (20 µg/mL). Equal number of cells (2 x 10^4^/well) with or without exosomes were added to each well and incubated for different time points (30–120 minutes) at 37°C. After each time point, attached cells were washed, fixed with cold methanol for 15 minutes and stained with 0.5% crystal violet dissolved in 25% methanol for 10 minutes. The plates were washed in water, dried overnight (12–16 hours) at room temperature and photographed using a DCM200 digital camera equipped with Scopephoto software. To determine the role of secreted exosomes in subsequent cellular attachment, the cells were pre-incubated with 30 µM BAPTA-AM for periods ranging from 1 to 7 h in culture medium. Studies have shown that pre-treatment of cells with BAPTA-AM down-regulates the secretion of exosomes. The controls were incubated in culture medium containing the same concentration of vehicle (DMSO) used to dissolve the BAPTA-AM. After the incubation, the cells were detached using 2 mM EDTA, washed in serum free medium and added to wells coated with 1% fetuin-A in the absence or presence of purified cellular or serum exosomes. We have shown that breast tumor cells bind rapidly to wells coated with fetuin-A in SFM and that fetuin-A is the major adhesion protein in serum [12].

### Live cell imaging of exosomes secretion and uptake

Live-cell imaging was performed at 37°C by using a 60X oil objective (numerical aperture, 1.4) of a laser scanning or Nikon A1R confocal microscope. BT-CD63 cells were plated on 35-mm imaging dishes (MatTek, Ashland, MA) and cultured overnight in complete DMEM/F-12 medium. For live exosomal secretion studies, EDTA (final concentration of 2 mM) was added to the adherent BT-CD63 cells maintained at 37°C. Time-lapse images were captured using a 60X oil objective (numerical aperture, 1.4) in the GFP channel at 5 second intervals using the laser scanning Nikon A1R confocal microscope. All movies were edited using Nikon EZ-C1 Free Viewer and saved as TIFF format for presentation in QuickTime. To study the exosomal co-trafficking with either cholera toxin B (CTXB) or transferrin (Tf), exogenous GFP-CD63-labeled exosomes (10 µg/ml) diluted in HBSS containing 0.5% BSA and 1 mM MgCl_2_ and CaCl_2_, were added directly to the culture medium which was pre-cooled on ice for 10 minutes. Alexa Fluor tagged CTXB (10 µg/ml) or Tf (10 µg/ml) were added to the culture medium and the cells were left on ice for an additional 10 minutes. This mixture was then brought to room temperature and imaged using the laser scanning Nikon TE2000 confocal microscope.

### Fluorescence recovery after photo-bleaching

BT-549 cells stably transfected with GFP-ANXA2 or GFP-ANXA6 were plated on glass coverslips in complete DMEM/12 (Invitrogen). After 24 h cells were washed three times with PBS, loaded or depleted of cholesterol, or mock treated for controls. To deplete the cells of cholesterol, the cell culture medium was remove and discarded, and cells were washed with PBS and incubated in HBSS 0.5% BSA containing 10 mM MβCD for 10 min. at 37°C. To load the cells with cholesterol, water-soluble cholesterol mixed with MβCD in a 1∶6 ratio (10 mM in MβCD) was added to warm HBSS containing 0.5% BSA for 10 minutes at 37°C. Cells were rinsed with PBS prior to imaging. Fluorescence recovery after photo-bleaching (FRAP) was performed on a Nikon A1R laser scanning confocal. We used a Plan-Apo 60×1.4 numerical aperture (NA) oil DIC lens at a zoom of 4x. GFP was excited with the 488 nm line of a 40 mW argon laser at 0.5–1% attenuated transmission and fluorescence emission collected using a 505 long pass filter. The pinhole was set to 1 Airy unit and no line averaging was used. We defined a 5 µm wide circular region of interest (ROI) and photo-bleached GFP with 250 ms dwell time at 100% transmission of a 40 mW argon laser. Since diffusion was very rapid for GFP-AnxA2 and GFP-AnxA6 in control conditions, we captured 15 images/s to produce our FRAP curves. FRAP recordings were done on cells in PBS, pH 7.4 at room temperature.

### Western Blot Analysis

Cells were grown in 15-cm dishes until 80–90% confluent in complete DMEM/F-12. The cells were washed once in ice-cold PBS and harvested by scraping. Cells were disrupted in RIPA buffer (50 mM Tris-HCl, pH 7.4, 1% Nonidet P-40, 0.1% sodium deoxycholate, 150 mM NaCl, 1 mM EDTA) containing a cocktail of proteases (Sigma) and phosphatase inhibitors (20 mM sodium fluoride, 50 mM β-glycerophosphate, and 1 mM sodium orthovanadate). Cell lysates or various fractions of sucrose gradients were separated in 4–12% SDS–PAGE (Invitrogen), blotted on Immobilon membranes (Southern Scientific). Blots were probed with the indicated primary antibodies followed by HRP-conjugated secondary antibodies and revealed using enhanced chemiluminescence. The intensity of the bands was quantified using ImageJ (NIH).

## Results

### Quantification of exosomal secretion by using GFP-CD63 as readout

**Figure 1:**
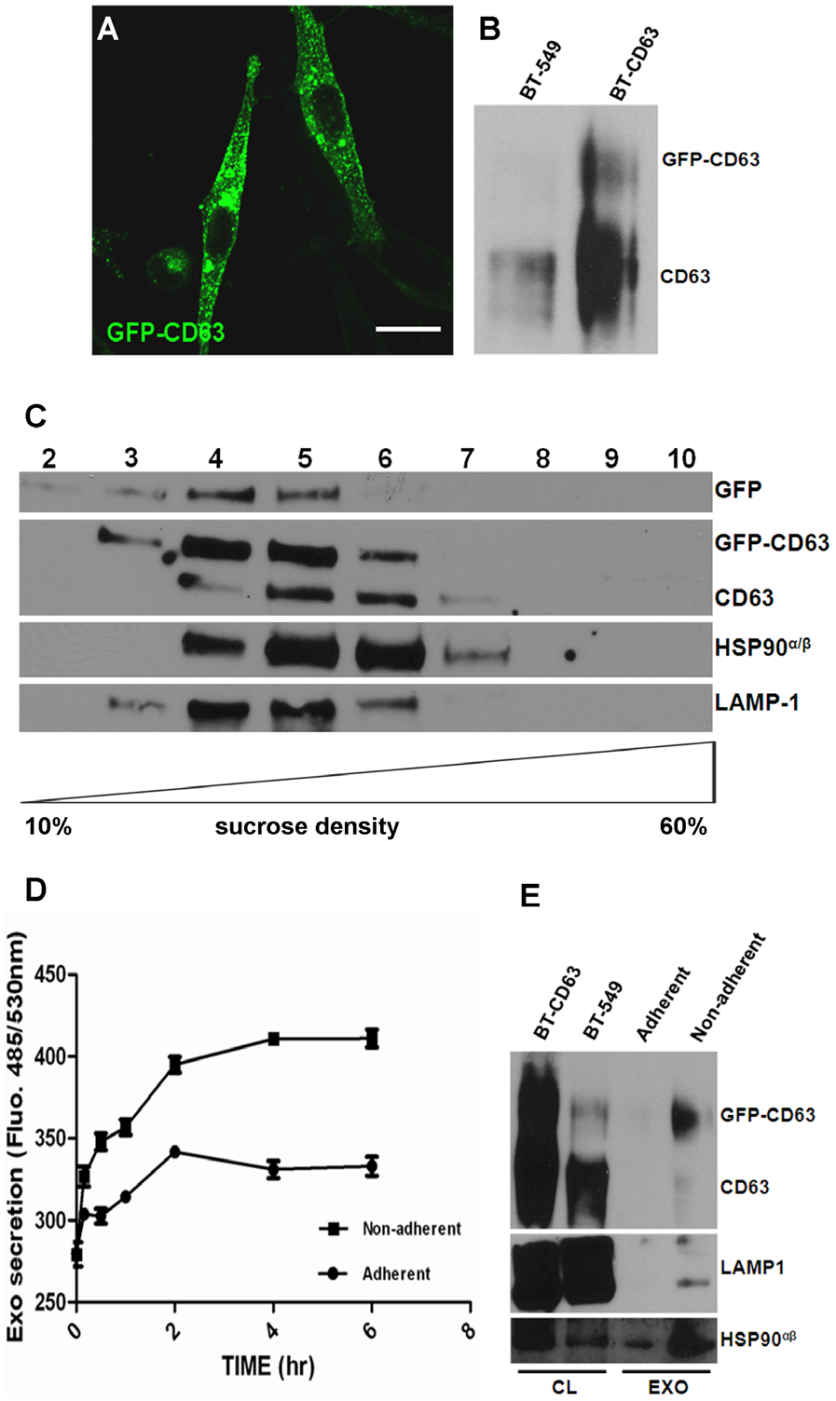
Detection and quantification of intracellular and released exosomes in the conditioned medium of BT-CD63. (A) BT-549 cells stably expressing GFP-CD63 were grown on glass cover-slips for 24 hours and the intracellular localization of the CD63-associated vesicles visualized by confocal microscopy. Bar is 10 µm. (B) Over-expression of GFP-CD63. Total cell lysates from control BT-549 and BT-549 expressing GFP-CD63 (BT-CD63) cells were analyzed by western blotting and probed with antibodies against CD63. (C) Exosomes purified from the spent media of BT-CD63 cells were layered on top of a sucrose step gradient and centrifuged at 200,000 x g for 4 hours in a Sorvall M150 microcentrifuge. Equal volumes of fractions collected from the top of the gradient (20 µL) were separated by SDS-PAGE and analyzed by western blotting for the co-migration of CD63 and the indicated exosomal markers. (D) BT-CD63 cells (5×10^6^) were cultured in complete medium for 24 hours. Adherent cells were maintained in phenol red and exosome-free DMEM/F-12 for the indicated times (0–6 hours). For non-adherent cultures, EDTA-detached cells were resuspended in phenol red and exosome-free DMEM/F-12 for the indicated times (0–6 hours). GFP-CD63 tagged exosomes in the conditioned media were assayed by fluorescence spectroscopy. (E) Purified exosomes secreted for 2 hours from adherent or non-adherent BT-CD63 cells (5×10^7^) were separated in 4-12% SDS-polyacrylamide gels and analyzed by western blotting using antibodies against CD63 and the indicated exosomal markers.

To study the mechanisms of exosomal secretion and uptake we have established a cell line (BT-CD63) stably expressing GFP-CD63 as a model for our studies (Fig. 1A and B). CD63 is a member of tetraspanins, a family of cell-surface associated proteins characterized by four transmembrane domains [25]. CD63 predominantly co-localize in the membrane of MVB/late endosomes and exosomes and give a characteristic punctate pattern on direct or indirect immune-fluorescence images [25]. Exosomes isolated and purified from the BT-CD63 cell line are decorated with GFP-CD63. GFP-CD63 is localized with other exosomal marker proteins, HSP90^α/β^ and LAMP-1 in fractions 4–6 of the 10-60% sucrose gradient (Fig. 1C). It is interesting to note that exosomal proteins usually elute in this range (fractions 4-6 of the 10–60% sucrose gradient) [29], [30]. Armed with the knowledge that the breast cancer cells that express GFP-CD63 secrete exosomes decorated with this protein, we obtained conditioned medium (1 ml) from attached and an equal number of detached BT-CD63 cells. Determination of GFP levels in the conditioned medium revealed that detached cells release more exosomes over a six hour period than the attached cells (Fig. 1D). The level of exosomal proteins secreted into the conditioned medium of adherent BT-CD63 cells for 2 h was compared to the level of proteins in the exosomes released by the same number of cells suspended for 2 hours. Results show that cells secrete approximately 5 to 6 times more exosomes when suspended for 2 h compared to the secretion over 16 hours period from the same number of cells that are attached (Fig. 1E).

### Cell detachment triggers a rapid release of exosomes

**Figure 2:**
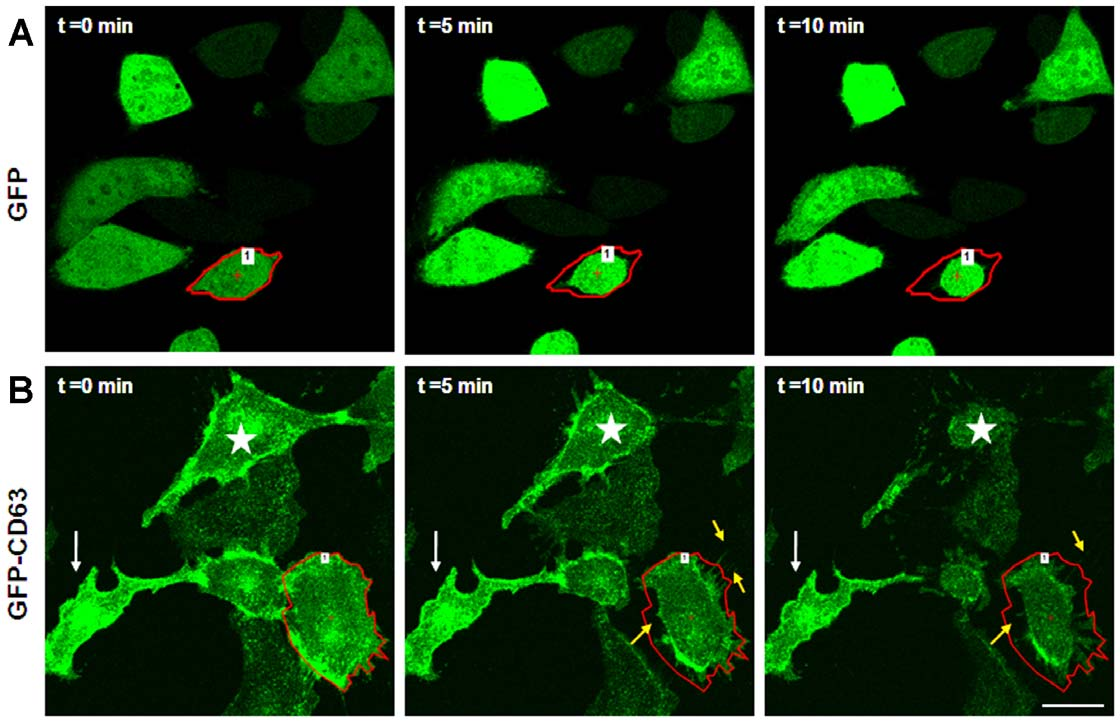
Visualization in real time of cellular detachment-induced rapid release of exosomes from BT-CD63 cells. BT-CD63 cells or GFP expressing control cells were plated on MatTek glass dishes for 24 hours. Prior to live-cell imaging cells were washed 2 times in PBS. Cells were then treated with EDTA (2 mM) and the trafficking and release of GFP-labeled exosomes was monitored by time-lapse imaging for up to 10 minutes. Upper panels are representative individual frames from time-lapse acquired images of GFP expressing control cells. Lower panels are representative individual frames tracking the release of the GFP-tagged CD63 vesicles. The white arrow shows a strongly adhered cell that was not detached even after 10 minutes following EDTA addition. The yellow arrows indicate stream of GFP-tagged CD63 vesicles that are being released (see movie S1 and S2 in supplemental materials). Bar is 20 µm.

**Figure 3:**
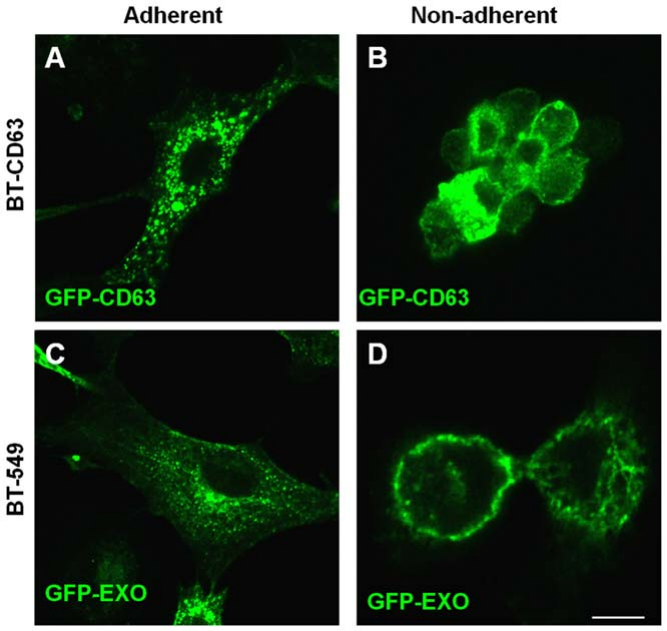
Distribution of GFP-CD63 containing exosomes in adherent and non-adherent BT-CD63 cells. A-B) BT-CD63 cells were maintained in adherent (panel A) or non-adherent (panel B) modes as described in materials and methods and examined by confocal microscopy Note that the EDTA-induced detachment of BT-CD63 led to the accumulation of the GFP-CD63 labeled exosomes on the cell surface. C-D), parental BT-549 cells in adherent cultures or in anchorage-independent cultures on polyHEMA coated plates were incubated with conditioned medium containing GFP-CD63 vesicles for 3 h. Cells were thereafter processed for microcopy as described in Materials and Methods. Bar is 10 µm.

To directly visualize the secretion/release of exosomes in live cells, we again employed GFP-CD63 expressing BT-CD63 cells. The cells were cultured on glass bottom culture dishes for 24 hours. Just prior to addition of EDTA (t = 0), cells showed what appears to be a background movement and release of GFP particles (Fig. 2B; movie S2). The addition of EDTA caused the cells to retract and concomitantly a stream of green particles could be seen shooting outwards within seconds (Fig. 2B; movie S2). The unidirectional movement of the exosomal particles could be seen going past the original cell borders marked by red. (Fig.2B). Following their release into the extracellular milieu, the particles (too small to be seen individually under microscope) dissipate and fade away, suggesting that they move as a group which then breakout as individual particles outside the cell. The exit of exosomes is clearly evident in this cell (marked by red boundary) as well as the cell marked by the star in Fig. 3B. To control for fluorescence bleaching with time, we also examined a GFP labeled epithelial cell within the time frames (see cell with red boundary) (Fig. 2A). Also as this control cell retracts and detaches upon the addition of EDTA, no particles are seen being secreted outwards (Fig. 2A; movie S1). This live cell imaging confirmed our observation that detachment accelerates the secretion of exosomes. We next wanted to determine the localization of exosomes in attached or adherent and spread cells as well as in non-adherent spherical cells. Whereas we and others have observed that in attached and spread cells, exosomes are concentrated in the perinuclear region of the cell [6], [18], the manner in which they re-equilibrate in detached spherical cells had not been described. As shown in Fig. 3, panels B and D, both endogenous and exogenous or donated GFP-CD63 containing exosomes are concentrated on the surfaces of non-adherent spherical cells. We have confirmed this observation using purified exosomes labeled with rhodamine isothiocynate (unpublished data). Endogenous GFP-CD63 containing exosomes can be seen concentrated in the perinuclear region in adhered and spread BT-CD63 (Fig. 3A). Like-wise, purified exosomes bearing GFP-CD63 added to attached and spread parental BT-549 enter the cells and localize to the perinuclear region of the cell (Fig. 3C).

### Exosomes promote adhesion and cell spreading to plastic, laminin and fibronectin

**Figure 4:**
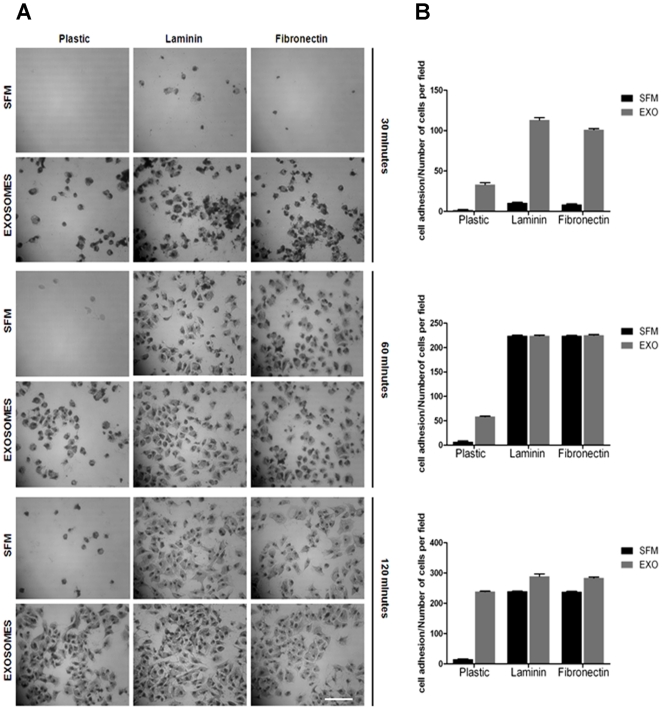
Exosomes promotes rapid adhesion of BT-549 breast cancer cells. The wells of a 96-well culture plate were either uncoated (plastic), or coated with 10 µg/ml of fibronectin or laminin for 16 hours at 37°C. Prior to use, the wells were washed twice with HBSS. BT-549 cells were pre-incubated in serum-free medium for 2 hours to deplete endogenous exosomes. The cells in serum free medium (SFM) were divided in 2 groups and re-suspended in SFM containing 1 mM Ca^2+^/Mg^2+^ without or with cell-derived exosomes (10 µg/ml). Cells (2×10^4^) were transferred to 96-well plates for indicated times (30–120 minutes) and incubated at 37°C. The unattached cells were aspirated with the SFM and the adhered cells fixed in cold methanol and stained with crystal violet. Cells were photographed and the number of attached cells assessed by cell counting. Each bar represents the mean ± S.E of adherent cells per field. Bar is 40 µm.

We previously showed that in addition to mediating anchorage independent growth of breast tumor cells, exosomes also promoted the adhesion of these cells to plastic [6]. Here we determine whether these vesicles also play a role in the rapid adhesion of tumor cells to the common extracellular matrix proteins such as laminin and fibronectin. The breast carcinoma cell line BT-549 adheres slowly to various substrata and can take as many as 6 hours to fully adhere to plastic and a minimum of 1 hour to laminin coated dishes [31]. We herein show that in the absence of cellular exosomes, cells failed to adhere to any substratum within the first 30 minutes, while in the presence of added exogenous exosomes (∼20 µg/ml), an appreciable number of cells adhered and spread on plastic (control), laminin and fibronectin coated wells within 30 minutes (Fig. 4, panels A and B). In the absence of exosomes, even after 2 hours of incubation, only a handful of cells adhered and spread on plastic (Fig. 4, panels A and B). Generally these cells require an overnight incubation to fully adhere to plastic.

### The significance of cell surface immobilized exosomes for subsequent adhesion and spreading

**Figure 5:**
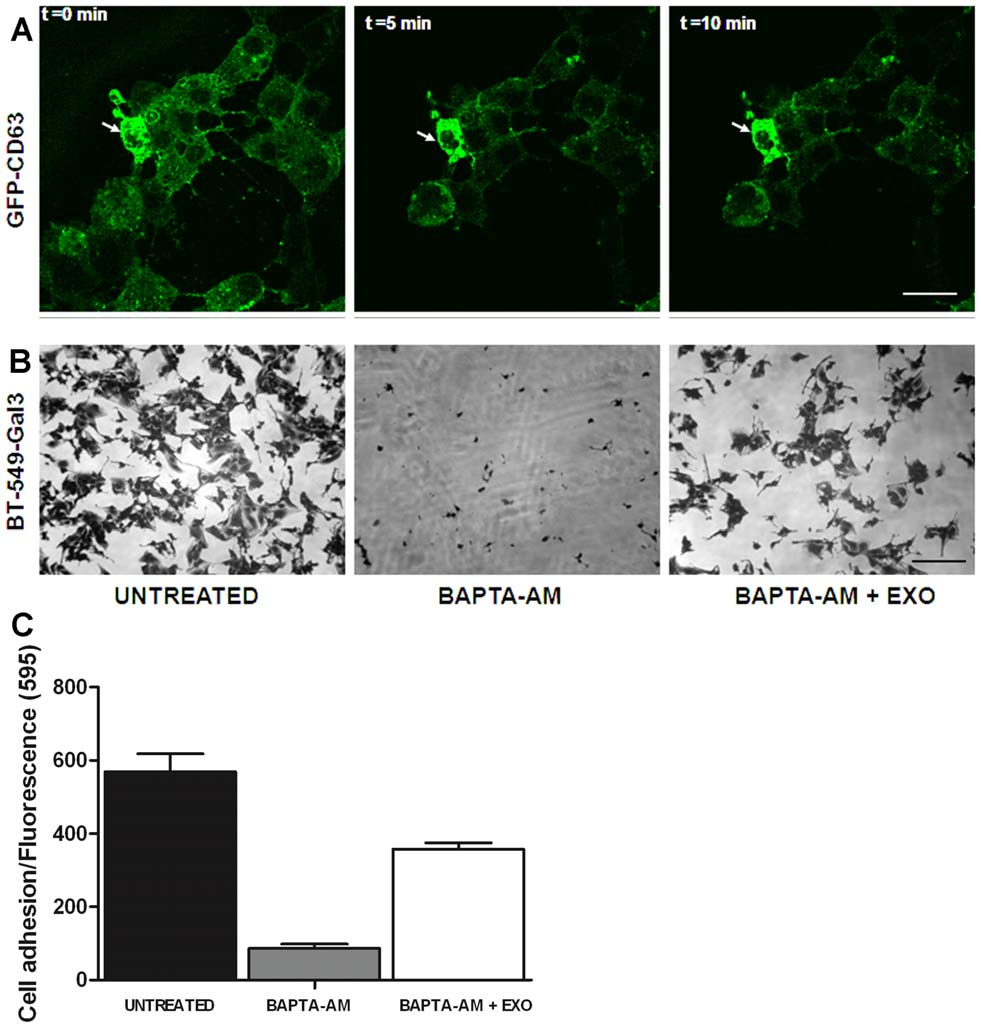
Exosomes secreted by tumor cells during detachment are required for subsequent adhesion and spreading. In panel A, BT-CD63 cells were plated in 35 mm with glass bottom dishes and allowed to adhere for at least 24 hours in complete DMEM/F-12 medium. The culture medium was replaced with fresh medium containing 30 µM BAPTA-AM and allowed to incubate for another 7 hours. The dish was then placed on microscope stage (Nikon A1R) and the live cells photographed (t = 0) and then 2 mM EDTA added. After 5 and 10 minutes of EDTA addition, images were again taken of the cells. Bar is 20 µm. In panel B, BT-549-Gal-3 cells in control wells (untreated) and in BAPTA-AM treated wells (in the absence and presence of purified cellular exosomes) were allowed to adhere to Fetuin-A coated wells, fixed, stained and photographed as described above. Bar is 40 µm. In panel C, the experiments in B were repeated with a different cell line (MDA-MB-231). At the end of the incubation, the non-adhered cells were washed off and fresh medium containing a 1∶10 dilution of Alamar Blue added to the wells, incubated for another 1 h and cell number determined by fluorescence spectroscopy.

We questioned whether the rapid secretion of exosomes and their localization on the cell surface is required for subsequent adhesion to the substrata. The release of exosomes is highly dependent on increases in intracellular [Ca^2+^] [15]. Pre-incubation of BT-CD63 with BAPTA-AM inhibited the sudden release of exosomes in live cells even after 10 min of addition of EDTA and so there was no accumulation of exosomes on the cell surfaces (Fig. 5A). To directly test this effect of BAPTA-AM on the subsequent adhesion of cells, we pre-incubated BT-549-Gal3 cells with BAPTA-AM for 7 hours prior to detachment following the addition of EDTA. The BAPTA-AM pre-treated cells were not able to adhere to fetuin-A coated wells even after 2 hours of incubation while the control (untreated) cells were fully adhered after only one hour (Fig 5B). Interestingly, the BAPTA-AM treated cells were able to adhere and spread on fetuin-A coated wells in the presence of 20 µg/ml of exosomes purified from the BT-549 cells (Fig. 5, panels B). To show that the release of exosomes for adhesion is not unique to BT-549-Gal3, we also treated MDA-MB-231, another popular breast cancer cell line without (control) and with 30 µM of BAPTA-AM for 1 h and demonstrated that whereas the control untreated cells adhered to fetuin-A coated wells after detachment, the cells pre-treated with BAPTA-AM failed to do so after detachment and only did so in the presence of added serum exosomes (125 µg/ml) (Fig. 5C). The serum exosomes were not as efficient as cell derived exosomes in promoting adhesion and a much higher concentration was used. The MDA-MB-231 cells appeared to be very sensitive to BAPTA-AM and so were incubated for 1 h. Only cells that were 100% viable after BAPTA-AM treatment were used for subsequent attachment studies. Therefore, flooding of cell surfaces with exosomes secreted upon detachment is critical for subsequent adhesion of cells particularly in serum free conditions.

### Uptake of exosomes via lipid rafts

**Figure 6:**
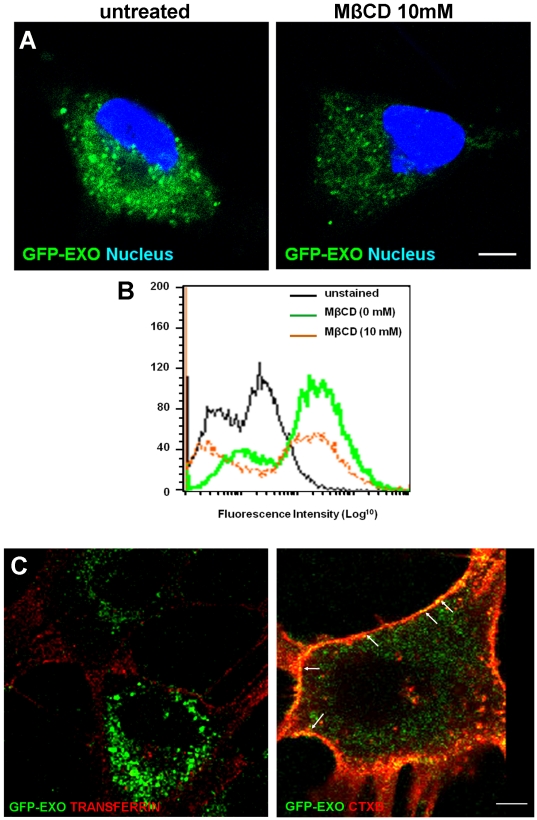
The exosomal uptake route in breast carcinoma cells. BT-549 cells were grown on glass cover-slips for 24 hours. Cells were then washed twice with HBSS, and treated with different concentrations of methyl-beta-cyclodextrin (MβCD) (0–10 mM) for 30 minutes. Subsequently, cells were incubated with 10 µg/ml GFP-CD63 labeled exosomes in HBSS containing 0.5% BSA and 1 mM Ca^2+^/Mg^2+^ for 30 minutes at 37°C. In panel A, the untreated (control) and cells pre-incubated with 10 mM of MβCD were washed in ice-cold HBSS and fixed with paraformaldehyde and cover-slips mounted with Prolong Gold and visualized by confocal microscopy. Images were captured using a Nikon TE2000. Bar is 20 µm. In panel B, the cells were also processed for flow cytometry as indicated. Unstained control cells (black line) were included. In panel C, BT-549 cells grown on MatTek glass bottom culture dishes were incubated in HBSS 0.5% BSA 1 mM Ca^2+^ 1 mM Mg^2+^ on ice for 10 minutes. The cells were then treated with 10 µg/mL of alexa fluor Tf (left panel) or CTXB (right panel) and GFP-labeled exosomes on ice for 10 minutes again. The trafficking of GFP labeled particles and colocalization with either Tf or CTXB (white arrows) were tracked live on the Nikon TE2000 for 10 minutes. Representative time-lapse images are shown.

The cellular mechanisms responsible for the uptake of exosomes have been the subject of considerable debate in the literature [32], [33]. We hypothesized that exosomes are internalized via the same gateway in the plasma membrane through which they are secreted which in most cases has been shown to be lipid raft domains [34]. Treatment of cells with methyl beta-cyclodextrin (MβCD) in the range of 0–10 mM was quite effective in reducing the uptake of GFP-CD63 labeled exosomes as indicated by reduced fluorescence (Fig. 6, panels A and B). To further show that the uptake of exosomes is via lipid rafts, GFP-exosomes were incubated with the cells in the presence of either alexa fluor tagged transferrin or cholera toxin B (CTXB) which are internalized via clathrin coated pits or lipid rafts respectively. The data show that GFP-exosomes co-migrate or traffic with CTXB (see arrows) but not transferrin, again suggesting that uptake of exosomes is via lipid rafts.

### Trafficking of exosomes in tumor cells

**Figure 7:**
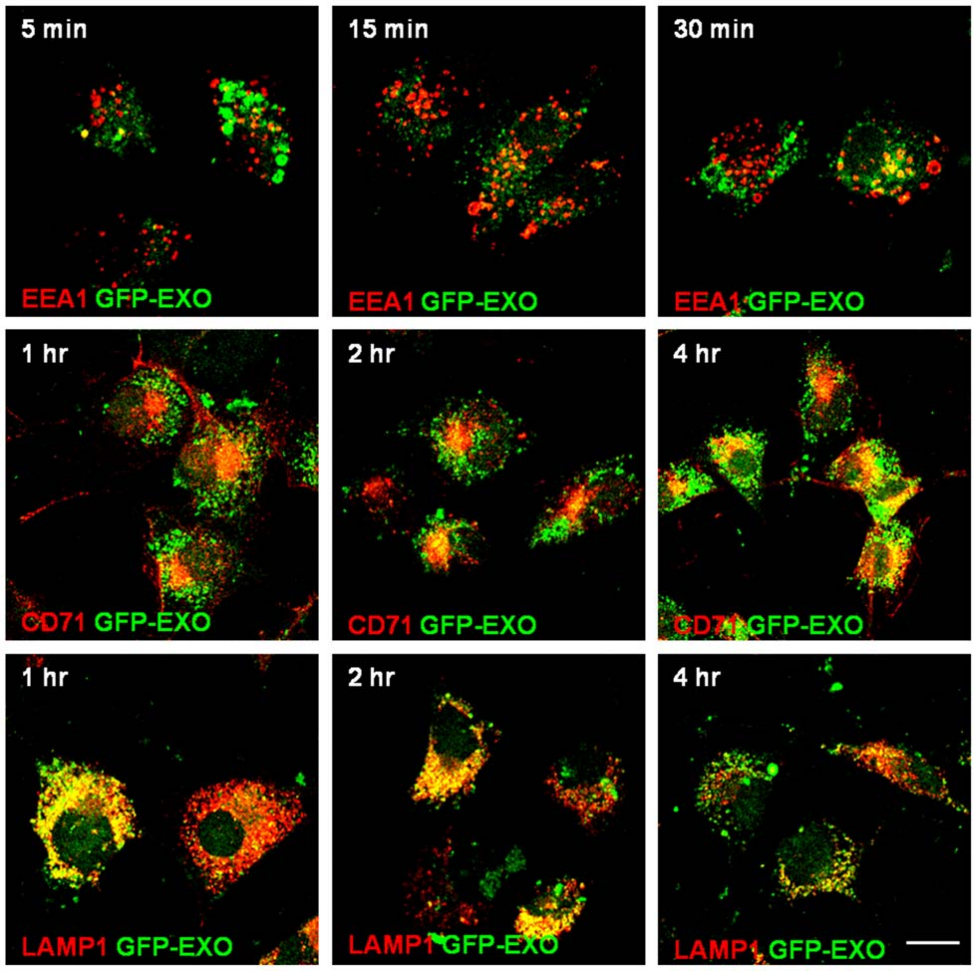
Trafficking of GFP-exosomes in breast carcinoma cells. BT-549 cells gown on glass cover slips were incubated with purified GFP-CD63 exosomes (green) for the indicated time points. At each time point the cells were fixed with PFA and stained with antibodies to the early, late and recycling endosomes followed by TRITC conjugated secondary antibodies (red). The first row shows co-localization of GFP-exosomes with EEA1 (early endosomes); Second row shows colocalization with CD71 (recycling endosomes); and the third row shows colocalization with LAMP1 (late endosomes). Bar is 10 µm.

In order to follow the uptake of exosomes more closely, we examined whether they localize with markers of early endosomes (EEA1), recycling endosomes (CD71) or late endosomes (LAMP-1) in a time dependent manner. For the first 15 minutes of incubation, the GFP-exosomes were hardly observed in early endosomes (Fig. 7). However, after 30 min, some colocalization with EEA1 was evident. After 1 h, GFP-exosomes were in the late endosomes where they stayed for at least another 1 h and then moved to the recycling endosomes after 4 h (Fig. 7).

### Annexins mediate the uptake of exosomes by tumor cells

**Figure 8:**
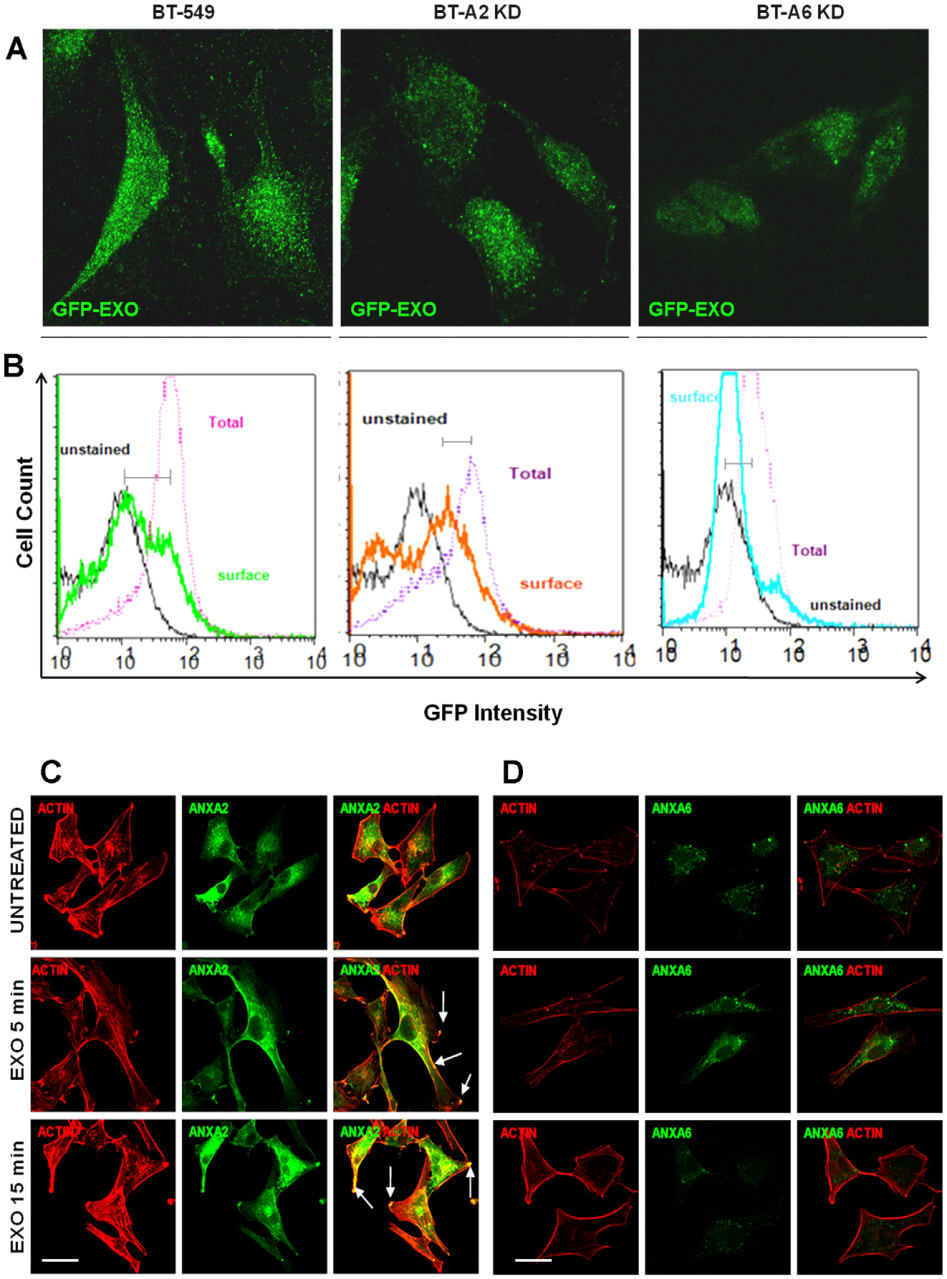
Annexin-mediated uptake and disposition of exosomes on the cell surface. Parental BT-549, BT-A2-sh and BT-A6-sh were grown on glass coverslips or 10 cm dishes and incubated with GFP-CD63 exosomes and analyzed by (A) confocal microscopy or (B) Flow Cytometry. In panels C and D, BT549 cells were grown on glass cover-slips, serum starved for 24 hours washed and stimulated without (control) and with 100 µg/mL exosomes in HBSS 0.5% BSA 1 mM Ca^2+^ 1 mM Mg^2+^ for 5 and 15 minutes at 37°C and fixed with PFA. Cells were then treated with rhodamine phalloidin for actin staining followed by antibodies to AnxA2 (C) and AnxA6 (D). Images were acquired with a Nikon TE2000 confocal microscope. Bar is 20 µm.

We have demonstrated that AnxA2 and AnxA6 are important for cellular adhesion and growth in culture [12]. Knowing that in adhered and spread cells exosomes are sequestered inside the cells, we questioned whether the annexins played a role in the uptake and intracellular trafficking of exosomes since these proteins are usually associated with cellular but not serum derived exosomes [6]. We therefore compared the uptake of GFP-CD63 bearing exosomes (GFP-EXO) in BT-549 and its sub-clones that have reduced expression of AnxA2 (BT-A2-sh) and AnxA6 (BT-A6-sh) (Fig. S1, supplemental data). There was a reduction in the uptake of exosomes (CD63-GFP) in BT-A2-sh and BT-A6-sh (Fig 8 A). The uptake of exosomes was quantified by comparing fluorescence intensities between surface and total GFP-CD63 fluorescence according to the method of Ostrowski et al. [28]. A big difference seen in parental BT-549 cells means a large uptake while a small difference means a smaller uptake as indicated by the horizontal lines (Fig. 8 B). Knowing that exosomes mediate adhesion of tumor cells to various substrata and that exosomes that mediate adhesion would be concentrated in the adhesion plaques, we questioned which annexin member would associate or co-localize with these plaques. The data show that it is AnxA2 that co-localized with actin at the adhesion plaques on the cell surface (arrows) (Fig. 8C), while AnxA6 was mainly localized in the cytosol (Fig. 8D).

### Fluorescence recovery after photobleaching (FRAP) assays

**Figure 9:**
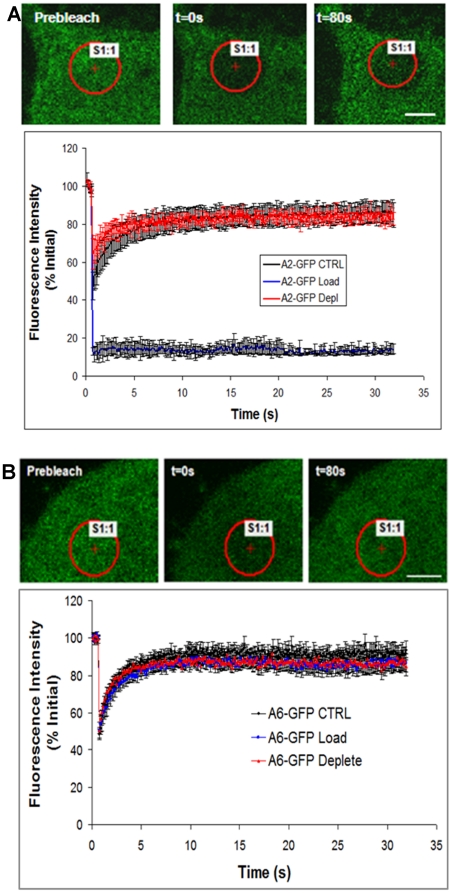
Fluorescence recovery after photo-bleaching. BT-549 stably transfected with either GFP-AnxA6 (panel A) or GFP-AnxA2 (panel B), were plated on glass coverslips in culture medium. After 24 h, the cells were washed 3X with PBS, loaded or depleted of cholesterol. Fluorescence recover after photo-bleaching (FRAP) was performed on a Nikon A1R laser scanning confocal microscopy as detailed in Materials and Methods. FRAP readings were done on cells in PBS, pH 7.4 and at room temperature.

Lipid raft membrane microdomains are cholesterol and glycosphingolipid-enriched [35]. Cholesterol is thought to play an important role in the structure and stabilization of lipid rafts and membrane proteins diffusing in lipid rafts have been shown to have an overall lower diffusion or mobility [36]. Proteins associated with caveolae-type lipid rafts on the cell surface are typically considered immobile [37]. To probe the dependence of AnxA2 and AnxA6 insertion within and diffusion on the plasma membrane, we removed (depleted) cholesterol with methyl-β-cyclodextrin (MβCD) and added (loaded) cholesterol using water-soluble cholesterol (MβCD–cholesterol complexes) in cells expressing these proteins. Cholesterol depletion in effect should allow a raft protein to diffuse faster, and under cholesterol loaded conditions, proteins would diffuse slower or be unable to diffuse if associated with caveolae [37]. AnxA2 had a slight but not significant increase in lateral mobility with cholesterol depletion, but was completely immobilized with cholesterol loading (Fig. 9A). We extended the recovery time after photo-bleaching for the AnxA2 in the loading condition to 3 min. We did not see any significant diffusion or mobility during this time frame as well (data not shown). These data suggest that AnxA2 is primarily associated with caveolae lipid rafts. We found that the lateral mobility of AnxA6 in cholesterol depleted or loaded conditions was not different from controls (Fig. 9B), suggesting that this family member is not associated with caveolae.

### To determine the annexins that move to the late endosomes in the absence and presence of added extracellular exosomes

**Figure 10:**
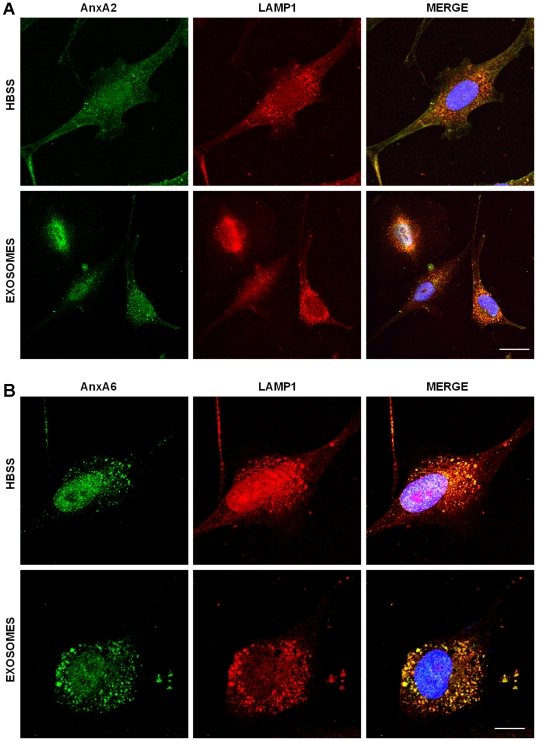
Trafficking of AnxA6 but not AnxA2 to the endosomal compartments as a function of exosomal uptake by BT-549 cells. BT-549 cells gown on glass cover slips were incubated without or with purified GFP-CD63 exosomes (green) for 2 h. The cells were then fixed with PFA and stained with antibodies to AnxA2 (panel A) or AnxA6 (panel B) followed by TRITC conjugated secondary antibodies (red). Bar is 10 µm.

The data from the FRAP experiments suggest that AnxA2 is immobilized on the cell surface while AnxA6 is mobile. In addition, the trafficking of exosomes in tumor cells showed the majority of exosomes translocated to the late endosomes. We therefore used the same GFP-AnxA2 and GFP-AnxA6 transfected breast carcinoma cells to determine which of these two family members is capable of translocating to the late endosomes in the absence and in the presence of added exosomes. AnxA2 did not move to the late endosomes (LAMP-1) after 2 h of incubation either in the absence or presence of added exosomes (Fig. 10A). However, AnxA6 was capable of migrating to the late endosomes after 2 h of incubation in the absence of exosomes. This trafficking to the late endosomes was substantially increased in the presence of added extracellular exosomes (Fig. 10B). Together, these data suggests that AnxA2 may be important in anchoring of exosomes to lipid raft domains of the plasma membrane while AnxA6 may be important in the trafficking of exosomes to the late endosomal compartment.

## Discussion

The present analyses were done to shed light on the mechanisms that mediate the secretion, endocytic uptake and the functional significance of exosomes in the extracellular milieu of tumor cells. A complete understanding of the mechanisms associated with exosomal mediated adhesion is essential towards the effort to corral metastatic cells, which are more likely to utilize this novel mode of adhesion. The data reported herein has enabled us to develop a working model to define exosomal mediated adhesion and growth related mechanisms.

We previously identified the ability of some tumor cells to adhere very rapidly to various substrata. More importantly we demonstrated that the tumor cells that adhered and spread rapidly also expressed high levels of galectin-3, suggesting that this lectin played a role in the rapid adhesion [31]. More recently we showed that the cells which express high levels of galectin-3 also rapidly secrete the lectin upon detachment from the substrata [24]. Based on current data, the detachment induces a spike in the level of intracellular calcium which in turn mediate the secretion of exosomes [15]. The process by which exosomes were secreted as observed in live cells was most intriguing. The secretory mechanism appeared to be novel due to its speed, in that almost 90% of exosomes were externalized within 10 minutes. The overarching finding from the present study, however, is that secreted exosomes that accumulate on the cell surface are needed for subsequent adhesion and growth of the tumor cells. Some of the exosomal proteins particularly galectin-3 and annexins have been shown to be up-regulated in tumor cells [38], [39], and hence the urgency to define their potential role(s) in the trafficking and utilization of exosomes by tumor cells.

The rapid adhesion of cells to the various substrata only in the presence of cellular exosomes, suggest that these vesicles harbor or carry an assortment of adhesion receptors including integrins [40]. The exosomes containing integrins and possibly other adhesion receptors are then likely to be anchored or immobilized at specific ‘adhesion plaques’ on the cell surface. The data suggest that tumor cells with the propensity to adhere rapidly to various extracellular matrix adhesion proteins and plastic, synthesize and secrete more exosomes relative to those that take longer to adhere and spread. This could be the domain of metastatic cells which have the capacity to rapidly adhere to various substrata to gain a foothold for growth advantage. Therefore as proof of principle, lack of secreted exosomes in BAPTA-AM treated cells following their detachment, mitigated their subsequent attachment and spreading. It is however unclear why nearly all of the exosomes in the cell have to be secreted outside only to be taken up by the cell during attachment and spreading. It is possible that exosomes transport adhesion receptors to the cell surface and once they have positioned the receptors, they need to be rapidly internalized for the receptors to function optimally. We have shown that in the presence of an overload of exosomes (>100 µg/ml) cells do not adhere and spread but instead form clumps (J. Ochieng, unpublished data). Another possibility is that exosomes may be critically needed for ‘initial’ adhesion which is then followed by stronger integrin mediated adhesion as in the rolling model of neutrophil migration into inflamed tissues [41]. We have previously documented the uptake of exosomes in breast carcinoma cells [6], while others have done so in other tumor cell types [42]. However, to date, there is no firm consensus as to the pathway by which exosomes enter or exit through the plasma membrane. The proposed mechanisms have ranged from entry via phagocytosis [18] to exit from the cells via cholesterol rich lipid rafts [43]. The present studies suggest lipid raft micro-domains as both the points of entry and immobilization of exosomes on the cell surface and that annexins particularly AnxA2 and AnxA6 play an active role in these processes.

Depletion of either AnxA2 or AnxA6 has been shown to attenuate cellular adhesion and spreading [12], [44], [45]. The immobilization of exosomes is likely to occur at the adhesion plaques concentrated in the lipid raft micro-domains, and these plaques associated with exosomes are likely to be responsible for the rapid cellular adhesion. AnxA6 on the other hand may be more relevant in the trafficking or re-cycling of exosomes from the cell surface into the cell and vice-versa via lipid raft domains. The present studies suggest important roles for AnxA2 and AnxA6 in exosomal mediated adhesion with implications in the progression of breast cancer. We have shown that silencing of AnxA6 abrogates cellular adhesion and spreading but promotes anchorage independent growth which may require exosomes to be concentrated on the cell surface [45]. Cellular adhesion and motility are not static processes; both involve the constant recycling of adhesion receptors [46]. Apart from mediating adhesion, exosomes interacting with their putative receptor(s) on the cell surface may elicit cell signaling. We have demonstrated that incubation of tumor cells with cellular or serum exosomes is sufficient to activate MAP kinase [6]. The localization of exosomes on the cell surface could also serve as platforms for cell-cell interaction such as homotypic aggregation [47].

In summary, this study shows that detachment of cells is a powerful trigger for the release of exosomes which are then concentrated on the surfaces of detached spherical cells. This release is likely to be a novel mechanism for shuttling adhesion receptors and possibly other signaling molecules to the cell surface. On the cell surface, exosomes interact with annexins where AnxA2 immobilize them on the cell surface at cholesterol rich lipid raft domains while AnxA6 mediates the uptake and recycling of excess exosomes via the same raft domains to facilitate adhesion and cell spreading.

## Supporting Information

Figure S1 **Knock-down and GFP-tagged AnxA2 and AnxA6 in breast carcinoma cells.** BT-549 parental cells were transfected with shRNA specific to AnxA2 (panel A) and AnxA6 (panel B). Stables puromycin-resistant clones were selected, expanded and depletion efficient was verified by Western blotting. BT-549 cells were transfected with GFP-AnxA2 (panel C) or GFP-AnxA6 (panel D) and the expression efficiency was verified by immunofluorescence and Western Blotting. Bar is 10 µm.
(TIF)

Movie S1 **GFP expressing control cells were plated on MatTek glass dishes for 24 hours.** Prior to live-cell imaging cells were washed 2 times in PBS. Cells were then treated with EDTA (2 mM) and the trafficking of GFP was monitored by time-lapse imaging for up to 10 minutes. GFP molecules remained inside the cells as they retract.
(M4V)

Movie S2 **BT-CD63 cells were plated on MatTek glass dishes for 24 hours.** Prior to live-cell imaging cells were washed 2 times in PBS. Cells were then treated with EDTA (2 mM) and the trafficking and release of GFP-labeled exosomes monitored by time-lapse imaging for up to 10 minutes. GFP-labeled exosomes shoot out as the cells retract and by the end of the movie, most of the GFP labeled particles have moved outside.
(M4V)
