# Supplementary figures and images for: Detachment of Breast Tumor Cells Induces Rapid Secretion of Exosomes Which Subsequently Mediate Cellular Adhesion and Spreading
Source: PLoS One. 2011 Sep 6;6(9):e24234. doi: 10.1371/journal.pone.0024234 (PMC3167827; doi:10.1371/journal.pone.0024234)

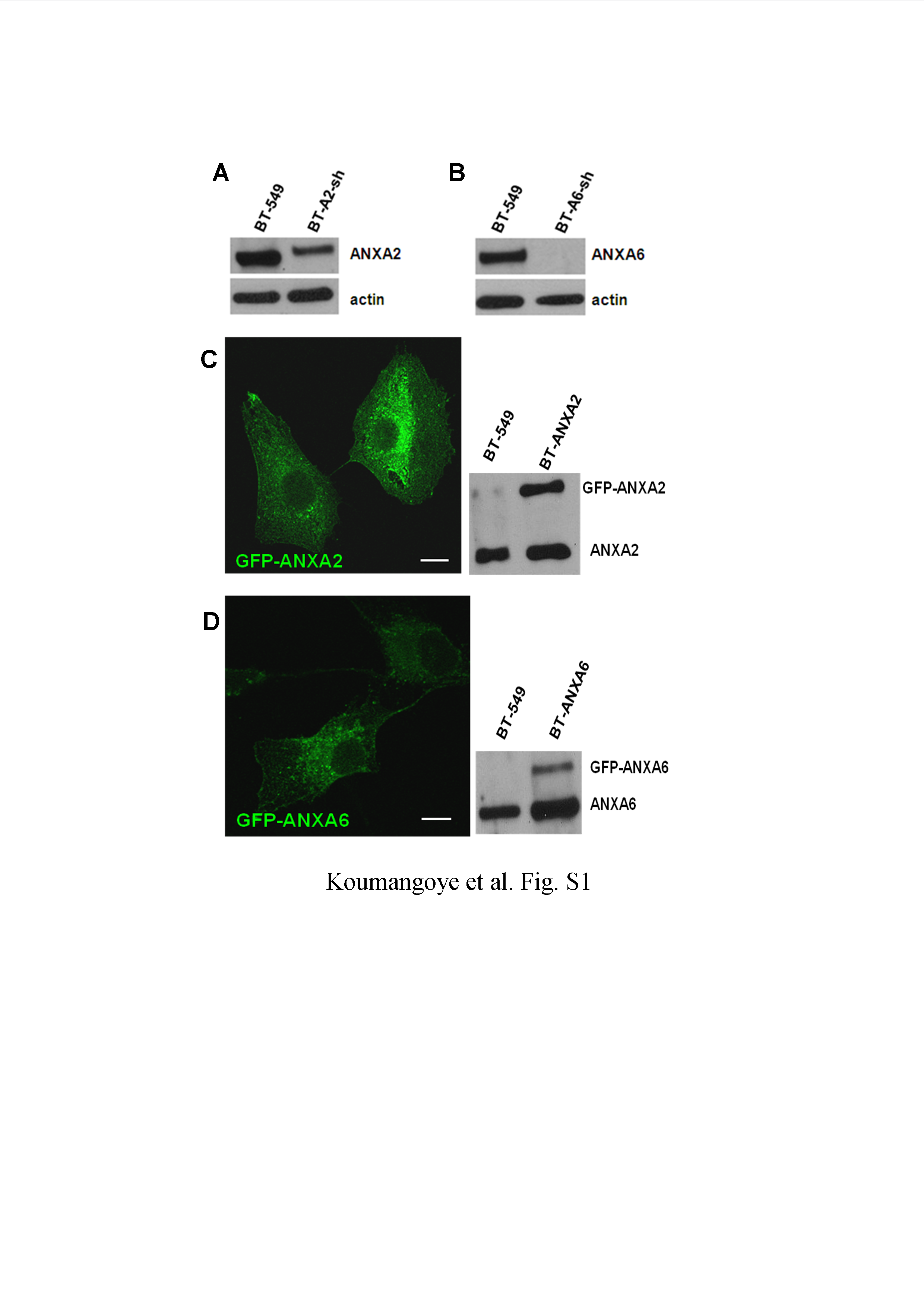

Supplement: Figure S1 — Knock-down and GFP-tagged AnxA2 and AnxA6 in breast carcinoma cells. BT-549 parental cells were transfected with shRNA specific to AnxA2 (panel A) and AnxA6 (panel B). Stables puromycin-resistant clones were selected, expanded and depletion efficient was verified by Western blotting. BT-549 cells were transfected with GFP-AnxA2 (panel C) or GFP-AnxA6 (panel D) and the expression efficiency was verified by immunofluorescence and Western Blotting. Bar is 10 µm. (TIF) [file pone.0024234.s001.tif]
